# Fumonisin B Determination in Maize Products from Belize Using an Immunosensor Based on Screen-Printed Carbon Electrodes

**DOI:** 10.3390/bios15080526

**Published:** 2025-08-12

**Authors:** Beatriz Pérez-Fernández, Britt Marianna Maestroni, Carlotta Cozzani, Colette Eusey, Natalie Gibson, Alfredo de la Escosura-Muñiz, Christina Vlachou

**Affiliations:** 1 NanoBioAnalysis Group, Department of Physical and Analytical Chemistry, University of Oviedo, Julián Clavería 8, 33006 Oviedo, Spain; bperfdez@gmail.com; 2 Food Safety and Control Laboratory, Joint FAO/IAEA Centre of Nuclear Techniques in Food and Agriculture, Department of Nuclear Sciences and Applications, International Atomic Energy Agency, Wagramerstrasse 5, A-1400 Vienna, Austria; b.m.maestroni@iaea.org (B.M.M.); c.cozzani@iaea.org (C.C.); c.vlachou@iaea.org (C.V.); 3 Belize Agriculture Health Authority, Central Investigation Laboratory, Belize City, Belize; colette.eusey@baha.org.bz (C.E.); natalie.gibson@baha.org.bz (N.G.); 4 Biotechnology Institute of Asturias, University of Oviedo, Santiago Gascon Building, 33006 Oviedo, Spain

**Keywords:** biosensor, fumonisin B, screen-printed carbon electrodes, point-of-need/use

## Abstract

A competitive electrochemical immunosensor, using screen-printed carbon electrodes (SPCEs), was developed for the determination of total fumonisins (sum of FB1, FB2 and FB3) extracted with a simple solvent extraction and dilution method, without clean up, from maize flour and maize tortillas. The optimized biosensor has a linear range of 0.25 to 50 µg/L with 3% and 2% reproducibility for FB1 and (FB1 + FB2), respectively, and a linear range of 0.25 to 10 µg/L with 2% reproducibility for (FB1 + FB2 + FB3). The limits of detection and quantification in PBS buffer for total fumonisins are 0.12 µg/L and 0.39 µg/L, respectively. These values in the maize matrix are 6.07 µg/kg and 20.25 µg/kg, respectively. In addition, the stability and the selectivity of the sensor were studied. The immunosensor was validated with liquid chromatography–tandem mass spectrometry. This novel biosensor is more rapid, simpler and cheaper than current methods, and can also be used at the point of need.

## 1. Introduction

Despite significant advancements in agricultural practices and food production technologies, mycotoxin contamination remains a persistent global challenge. Mycotoxins are toxic secondary metabolites produced naturally by specific fungal species and are estimated to contaminate at least 25% of the world’s agricultural commodities [1]. Contamination can occur pre-harvest, during crop cultivation and post-harvest, particularly during storage. A critical concern regarding mycotoxins is their thermal stability, which renders conventional food processing and cooking methods ineffective at reducing their concentration [2].

Fumonisins B (FB) are a group of mycotoxins primarily produced by Fusarium verticillioides and Fusarium proliferatum [3,4]. Among them, fumonisin B1 (FB1) is the most prevalent and has been classified as possibly carcinogenic to humans (Class 2B) by the International Agency for Research on Cancer (IARC) [5,6,7]. FBs exert their toxicity by inhibiting ceramide synthase, a key enzyme in sphingolipid biosynthesis. These toxins are highly hazardous to animals, causing equine leukoencephalomalacia in horses and pulmonary edema syndrome in pigs [8,9,10]. In humans, FBs may lead to acute gastrointestinal symptoms such as abdominal pain, rumbling and diarrhea, and chronic exposure has been associated with esophageal and hepatic cancers [11,12].

FBs are commonly found in maize and maize-derived food products. Due to their thermal resistance, FBs can persist through processing, posing a significant risk in widely consumed products such as maize tortillas. Given their toxicity, rigorous control of FB concentrations in food is essential to ensure compliance with food safety regulations. For instance, the European Commission has established maximum permissible levels of 1 mg/kg (sum of FB1 and FB2) in maize-based products for general consumption and 0.2 mg/kg in baby foods and products for infants and young children [13]. This regulation is particularly relevant in countries like Belize, where maize tortillas are staple foods, especially among children.

Chemically, FBs are highly polar and water-soluble compounds. FB1 is typically the most abundant, accounting for approximately 70% of total FB contamination, though FB2, FB3 and FB4 are also detected, albeit at lower levels [14].

The current gold standard for FB detection is high-performance liquid chromatography coupled with mass spectrometry (HPLC-MS) [15,16]. Other established methods include gas chromatography–mass spectrometry (GC-MS) [17,18], thin-layer chromatography (TLC) [19,20] and immunochemical techniques such as enzyme-linked immunosorbent assays (ELISAs) [21,22]. However, these methods often involve high costs, require skilled personnel and depend on large volumes of reagents. To overcome these limitations, electrochemical detection methods are increasingly explored for mycotoxin analysis due to their high sensitivity, cost-effectiveness, operational simplicity and minimal personnel training requirements. Several reviews have highlighted their growing importance in this field [23,24,25,26,27].

Electrochemical biosensors often incorporate specific recognition elements such as aptamers (aptasensors), antibodies (immunosensors) or enzymes (enzymatic sensors). In the case of FB detection, aptasensors [28,29] and immunosensors [30,31] are commonly used due to their high selectivity. Lateral flow-based sensors represent the most widely adopted format for FBs [32,33,34], but other electrochemical platforms such as indium tin oxide electrodes (ITO [35,36]), glassy carbon electrodes (GCEs [37]), and screen-printed carbon electrodes (SPCEs [38,39]) have also been employed. Table 1 summarizes the key analytical performance metrics of selected electrochemical sensors developed for FB detection.

Screen-printed carbon electrodes (SPCEs) have garnered considerable interest in the agri-food sector due to their versatility, compact size, sensitivity and suitability for on-site applications [40,41]. They have been successfully applied for the detection of various contaminants, including pesticides [42,43], mycotoxins [44,45,46,47,48,49], heavy metals [50,51,52,53] and drug residues [54,55,56].

Although SPCE-based immunosensors for FB detection have been reported, few studies have addressed the simultaneous detection of total fumonisins (FB1 + FB2 + FB3), and many require labor-intensive extraction protocols. Furthermore, validation of these sensors using standard analytical techniques is often lacking.

In this context, the objective of this study was to develop and validate an electrochemical immunosensor based on screen-printed carbon electrodes (SPCEs) for the detection of total fumonisins (FB1 + FB2 + FB3) in maize and maize-based food products. The method employs a simplified solvent extraction and dilution protocol and is validated against liquid chromatography–tandem mass spectrometry (LC-MS/MS).

**Table 1:** Analytical characteristics of different electrochemical immunosensors reported for fumonisin detection.

Fumonisin	Approach	Electrode	Linear Range (µg/L)	LOD (µg/L)	Ref.
FB1	Aptasensor	AuNPs/GS-TH/GCE	0.001–1000	0.001	[37]
FB1	Aptasensor	AuNPs/SPCE	0.5–500	0.14	[39]
FB1	Aptasensor	AuNPs/PDMS/SPCE	0.01–50	0.0034	[57]
FB1	Aptasensor	rMoS2-Au/GCE	0.001–100	0.0005	[58]
FB1	Aptasensor	PGE	10–40	3.69	[59]
FB1	Aptasensor	AuE	0.001–100	0.00026	[60]
FB1	Immunosensor	AuNPs/PPy-ErGO/SPCE	200–4500	4.2	[38]
FB1	Immunosensor	SWCNT/CS/GCE	0.01–1000	0.002	[61]
FB1, FB2	Immunosensor	SPGE	10–1000	5	[62]
FB1, FB2	Immunosensor	MBs/SPCE	0.73–11.2	0.33	[63]
FB1	Immunosensor	AuNPs/ITO	0.3–140	0.097	[64]
FB1	Immunosensor	SPCE	0.25–50	0.14	This work
FB1 + FB2			0.25–50	0.15	
FB1 + FB2 + FB3			0.25–10	0.12	

AuNPs: gold nanoparticles; PDMS: polydimethylsiloxane; SPCE: screen-printed carbon electrode; GS-TH: graphene/thionine nanocomposites; GCE: glassy carbon electrode; rMoS2-Au: co-reduced molybdenum disulfide and gold nanoparticles; PGE: pencil graphite electrode; AuE: gold electrode; SWCNT: single-walled carbon nanotubes; CS: chitosan; PPy: polypyrrole; ErGO: electrochemically reduced graphene oxide; SPGE: screen-printed gold electrode; MBs: magnetic beads; ITO: indium tin oxide.

## 2. Materials and Methods

### 2.1. Materials

Phosphate-buffered saline solution (PBS) 10 mmol/L, pH 7.4; bovine serum albumin fraction V (BSA) (Ref. A3059); fumonisin B1 solution (FB1; Ref. 34139); fumonisin B1 and B2 mixture (FBs-MIX) solution (FB1 + FB2; Ref. 34143); ochratoxin A (OTA; Ref. 34037) and ochratoxin B (OTB; Ref. 32411); zearalenone (ZEN certified reference material; Ref. CRM46916); aflatoxin Mix 4 solution (AF-MIX) (containing aflatoxin B1, B2, G1 and G2; Ref. 33415); deoxynivalenol (DON; Ref. 34124); and 3,3′,5,5′-Tetramethylbenzidine (TMB) (Ref. T0440) were purchased from Sigma-Aldrich (Taufkirchen, Germany). Fumonisin antigen conjugate (BSA-FB) (Ref. CJ-05-BSA) was purchased from Aokin (Berlín, Germany). Mouse monoclonal antibody specific to fumonisin (mAb-FB) (Ref. MBS190952) was purchased from MyBioSource (San Diego, CA, USA). Polyclonal rabbit anti-mouse IgG-HRP (anti-IgG-HRP) (Ref. P026002-2) was purchased from Drako, Agilent (Waldbronn, Germany). Fumonisin B3 solution (FB3; Ref. 32606–Supelco, Merck, Darmstadt, Germany); isopropanol (Ref. 1.01040.2500 Supelco, Merck, Darmstadt, Germany); formic acid (Ref. 1.00264.0100 Merck, Darmstadt, Germany); ammonium formate (99% crystalline, Alfa Aesar Gmbh & Co.Kg, Emmerich am Rhein, Germany); methanol (Ref. 14262); and acetonitrile (Ref. 14261) were purchased from VWR International GmbH (Wien, Austria). All chemicals employed were of analytical reagent grade. The 0.45 µm syringe filters (nylon) (Ref. 514-1279) and 0.2 µm syringe filters (nylon) (Ref. 514-1280) were purchased from VWR International GmbH (Wien, Austria).

A MilliQ Integral 3 purification system from Merck Millipore (Darmstadt, Austria) was used to obtain ultrapure water.

Working solutions of BSA were prepared in 10 mmol/L PBS pH 7.4, and BSA-FB, mAb-FB and anti-IgG-HRP were prepared daily in 10 mmol/L pH 7.4 PBS buffer with 0.5% BSA.

Working solutions of total FBs (FB1 + FB2 + FB3) were prepared daily from the initial stock standards in acetonitrile/water (1:1, *v*:*v*).

The maize used in this study was the common Zea Mays, also known as corn in the US, and was intended for human consumption. Organic canned maize kernels, used for method validation studies, were bought in an Austrian supermarket. The maize kernels were freeze-dried using an Alpha 2-4 LSCplus freeze dryer (Rieger Industrievertretungen GmbH, Wien, Austria). Original maize tortillas, made from local common maize flour, were sampled in tortilla shops in Belize. When referring to the origin Belize, it means that the maize and the tortillas were produced and purchased in Belize, geographical origin only, not of a specific species or variety. All samples, freeze-dried maize kernels and tortillas, were processed using a sample homogenizer, GM 200 (Retsch GmbH, Haan, Germany). Samples were prepared using a vortex mixer (IKA Werke, Staufen, Germany) and a Sigma 3-30 KS centrifuge (VWR International GmbH, Wien, Austria).

Chronoamperometric measurements were performed using an EmStat3 Blue potentiostat controlled by PSTrace 5.11 (PalmSens, Houten, The Netherlands), controlled by a tablet, with on screen-printed carbon electrodes (SPCEs) (Ref. DRP-110, from Dropsens (Oviedo, Spain)).

The method was cross-validated by analyzing the extracts from the maize flour, the maize kernels and the maize tortillas by ultra-high-performance liquid chromatography (Shimadzu Nexera X2 Series, Shimadzu Corporation, Kyoto, Japan), coupled to a Shimadzu 8060 triple quadrupole mass spectrometer (Shimadzu Corporation, Kyoto, Japan), consisting of an LD-30AD solvent delivery unit, an SIL-30AC autosampler, a CBM-20A communications bus module and a CTO-20AC prominence column oven. For the chromatographic separation, an Acquity UPLC^®^ BEH C8, 1.7 µm and 2.1 mm × 100 mm, column coupled to an ACQUITY UPLC BEH C8 VanGuard pre-column, 1.7 µm and 2.1 mm × 5 mm (Waters Corporation, Milford, MA, USA), was employed. The LC-MS/MS analysis conditions were as follows: mobile phase A: 0.1% *v*/*v* formic acid in water containing 10 mmol/L ammonium formate; mobile phase B: 0.1% *v*/*v* formic acid in methanol/water (9:1, *v*:*v*) containing 10 mmol/L ammonium formate. Gradient program: 0% B to 20% B over 2 min, 20% B to 100% B from 2 to 8 min, held at 100% B until 10 min, then decreased to 0% B. Total run time: 14 min with a flow rate of 0.3 mL/min. Extended rinsing of the system was performed over 6 min with 0.1% formic acid in methanol/water/isopropanol (1:1:1, *v*:*v*:*v*). Oven temperature: 40 °C. Injection volume: 2 µL. Mass spectrometry parameters and collision energies were carefully optimized to maximize analyte response. The nebulizing gas was set at a flow rate of 2 L/min, with both heating and drying gases maintained at 10 L/min. Instrument conditions included an interface temperature of 300 °C, a desolvation line temperature of 250 °C and a heat block temperature of 500 °C. Electrospray ionization (ESI) was performed in positive ion mode, applying an interface voltage of 4.0 kV. The multiple reaction monitoring (MRM) transitions and collision energies were selected and optimized by flow injection analysis. The fumonisins B, the optimized transitions and the collision energies chosen are summarized in Table S1 of the supplementary information.

### 2.2. Methods

#### 2.2.1. Preparation of Blank Maize and Tortilla Samples

Organic canned maize kernels and tortilla samples were freeze-dried and homogenized using a sample homogenizer for 30 s at maximum speed (10,000 rpm) at room temperature, using a space-reducer device. LC-MS/MS analysis was performed to confirm the absence of fumonisins in the homogenized maize flour.

#### 2.2.2. Preparation of Fortified Maize Samples

Homogenized maize sample portions weighing 5 g were enriched with an FB mixture (FB1, FB2 and FB3) at three concentration levels: 10, 100 and 200 µg/kg. The FB mixture was prepared from individual stock standards of FBs-MIX (FB1 + FB2) and FB3. The experimental conditions for each method validation study included five replicate analytical samples at each concentration level, with the entire study repeated on three separate days.

#### 2.2.3. Extraction of Fumonisins

**Figure 1:**
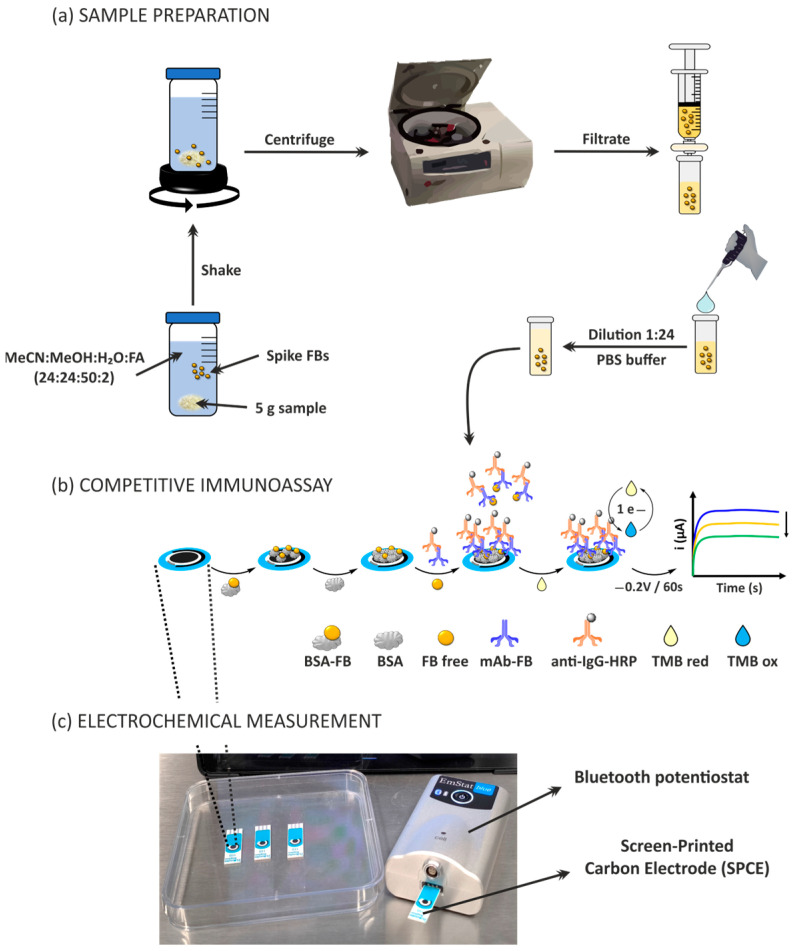
Scheme of (**a**) maize sample preparation for FB determination; (**b**) competitive immunoassay for total FBs on screen-printed carbon electrodes (SPCEs); (**c**) picture of the setup used for electrochemical measurements via Bluetooth connection.

Both blank and fortified maize and tortilla samples underwent extraction and dilution process [65,66,67], as shown in Figure 1a. A 5 g portion of homogenized maize was transferred to a 50 mL Falcon tube, to which 2 mL of MilliQ water and 10 mL of an extraction solution (acetonitrile/methanol/water/formic acid in a 24:24:50:2 ratio) were added. The tube was briefly shaken using a vortex mixer and subsequently placed on a shaker for 15 min at room temperature at 2500 rpm. After centrifugation at 10,000 rpm for 5 min at 5 °C, the supernatant (sample extract) was filtered through a 0.45 µm nylon filter and stored in a dark-labeled vial for further dilution. For LC-MS/MS injection, the sample extract was diluted with water and a generic solution (acetonitrile/methanol/water, 25:25:50) in a 4:2:4 ratio (water/generic solution/sample extract). Calibrators were similarly diluted.

Both calibrators and sample extracts were further diluted in a 1/24 ratio (sample extract/buffer) with the measurement buffer (10 mmol/L PBS pH 7.4; 0.5% BSA) before electrochemical detection using the SPCE. 

#### 2.2.4. Competitive Immunoassay for Fumonisin (FB) Detection

To immobilize the conjugated antigen (BSA-FB), 10 µL of a 25 µg/mL solution (in 10 mmol/L PBS, pH 7.4; 0.5% BSA) was placed on the SPCE working electrode and incubated for 90 min at room temperature (Figure 1b). The immobilization of the conjugated antigen on the working electrode is based on passive adsorption, mainly through hydrophobic interactions and electrostatic forces between the amino acid residues of the protein and the carbon surfaces. This immobilization method is commonly used in the development of biosensors using non-modified SPCEs. The non-adsorbed conjugate was removed by washing with ultrapure water. The electrode surface was blocked by immobilizing BSA from 40 µL of a 0.5% solution (in 10 mmol/L PBS, pH 7.4B) for 20 min at room temperature, followed by washing. Then, 20 µL of a solution containing 2 µg/mL monoclonal antibody (mAb-FB) and 10 µg/mL anti-IgG-HRP in 10 mmol/L PBS pH 7.4; 0.5% BSA (mixed and incubated during 15 min at 20 °C under stirring at 400 rpm) and the fumonisin solution (FB1, FB1 + FB2, or FB1 + FB2 + FB3) in 10 mmol/L PBS pH 7.4; and 0.5% BSA (concentration range 0.1 to 2000 µg/L) or fortified extracts was placed on the electrode and allowed to react for 60 min at room temperature. A washing step was carried out before the enzymatic reaction and electrochemical detection.

#### 2.2.5. Electrochemical Detection

An amount of 40 µL of TMB solution was applied to the electrode and incubated for 1.5 min, shielded from light. The analytical signal was recorded as the current at 60 s in chronoamperometric mode at an applied potential of −0.2 V, which corresponds to the reduction of TMB. Chronoamperometry was selected for its simplicity, sensitivity and compatibility with portable instrumentation. This is a standard method for the monitoring of the enzymatic reaction involving HRP tags and TMB substrate. The reduction potential of the oxidized TMB substrate was optimized in previous works [42,43,45], with a value of −0.2 V, showing better performance. Each measurement was performed in triplicate. A schematic of the electrochemical setup is shown in Figure 1c.

## 3. Results and Discussion

### 3.1. Evaluation and Adjustment of Key Parameters in the Development of a Fumonisin Immunosensor

The competitive immunoassay designed for the detection of total fumonisins (FB1 + FB2 + FB3) is schematically illustrated in Figure 1b. In this assay, competition occurs between the conjugated fumonisin (BSA-FB), immobilized on the working electrode’s surface, and the free FB analyte for binding to the monoclonal antibody (mAb-FB). Subsequently, the bound complex is recognized by the enzyme-labeled polyclonal antibody (anti-IgG-HRP). When the TMB substrate is added, horseradish peroxidase (HRP) catalyzes its oxidation. The oxidized TMB is subsequently electrochemically reduced by applying a potential of −0.2 V. The analytical signal is defined as the absolute value of the catalytic current recorded at the 1 min mark, which directly correlates with the concentration of FBs present in the sample.

**Figure 2:**
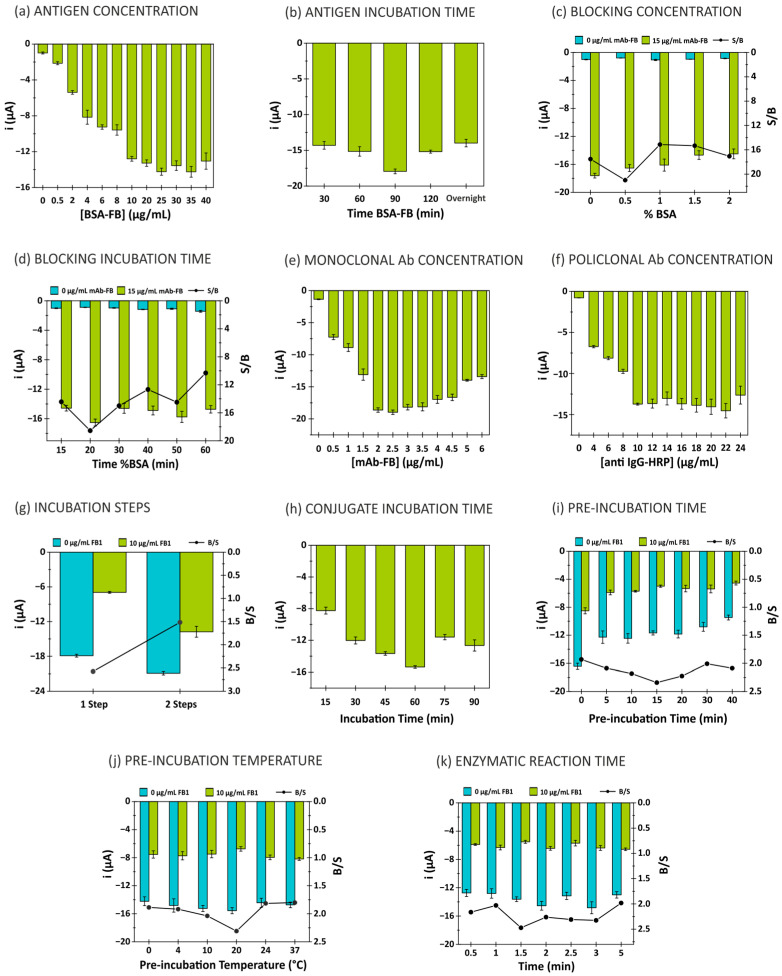
Evaluation of (**a**) BSA-FB concentration; (**b**) BSA-FB incubation time; (**c**) BSA concentration; (**d**) BSA incubation time; (**e**) mAB-FB concentration; (**f**) anti IgG-HRP concentration; (**g**) incubation steps; (**h**) immunoreaction time of BSA-FB–mAb-FB–anti IgG-HRP conjugate; (**i**) pre-incubation immunoreaction time; (**j**) pre-incubation immunoreaction temperature; (**k**) enzymatic reaction time.

A number of critical parameters affecting the analytical signal were systematically evaluated, including reagent concentrations, incubation times, enzymatic reaction time and electrode surface blocking (Figure 2). S/B, shown in some graphs, refers to the signal-to-blank ratio. This ratio is selected to evaluate the conditions in which the studied parameter produces an increase in the analytical signal. Conversely, in the studies where the competitive immunoassay is involved, as the studied parameter (incubation steps, pre-incubation time) produces a decrease in the analytical signal, the blank-to-signal ratio (B/S) is selected and shown.

#### 3.1.1. Evaluation of Antigen Conditions (BSA-FB)

The antigen (BSA-FB) was immobilized from solutions prepared at different concentrations (from 0.5 to 40 µg/mL) in 10 mmol/L PBS at pH 7.4 containing 0.5% BSA. The following experimental conditions were maintained throughout: 1% BSA incubated for 30 min; BSA-FB incubated overnight at 4 °C; 15 µg/mL mAb-FB (in 10 mmol/L PBS pH 7.4; 0.5% BSA); 20 µg/mL anti-IgG-HRP (in 10 mmol/L PBS pH 7.4; 0.5% BSA); 60 min of incubation of mAb-FB with anti-IgG-HRP at room temperature; 2 min of enzymatic reaction time. The analytical signal increased with the antigen concentration up to 25 µg/mL, beyond which no significant increase was observed. This concentration was selected as optimal, as shown in Figure 2a.

The incubation time for BSA-FB was optimized by testing durations of 30, 60, 90 and 120 min, and overnight incubation. A 90 min incubation was found to be optimal, as shown in Figure 2b.

#### 3.1.2. Evaluation of Blocking Agent Conditions (BSA)

To prevent non-specific adsorption, various concentrations of bovine serum albumin (BSA) were tested (0, 0.5, 1.0, 1.5 and 2.0% *w*/*v*). The other parameters were as follows: BSA-FB concentration of 25 µg/mL, BSA-FB incubation time of 90 min at room temperature, BSA incubation time of 30 min, mAb-FB at 15 µg/mL, anti-IgG-HRP at 20 µg/mL, and incubation times for both antibodies of 60 min at room temperature, with an enzymatic reaction time of 2 min. The optimal BSA concentration was selected based on the maximum absolute difference between the specific signal (with monoclonal antibody) and non-specific signal (without monoclonal antibody), which occurred at 0.5% *w*/*v* of BSA (Figure 2c). Negative controls without the primary antibody allowed for the evaluation of the background signal generated by non-specific binding or interferences. The incubation time for 0.5% BSA was optimized by evaluating durations of 15, 20, 30, 40, 50 and 60 min. A 20 min incubation was found to be optimal, as it provided the best ratio of non-specific to specific signal (Figure 2d), indicating effective blocking with minimal background interference.

These optimized blocking conditions significantly improved the signal-to-noise ratio (where the signal corresponds to the presence of the primary antibody and the blank refers to its absence), contributing to a lower detection limit and enhanced reproducibility of the immunosensor.

#### 3.1.3. Evaluation of Monoclonal Antibody Concentration (mAb-FB)

The concentration of monoclonal antibody (mAb-FB) was optimized in the range of 0.5 to 6 µg/mL, with the immunoassay time set to 1 h. The optimal concentration of mAb-FB was found to be 2 µg/mL, as shown in Figure 2e.

#### 3.1.4. Evaluation of Polyclonal Antibody Concentration (Anti-IgG-HRP)

The concentration of the enzyme-labeled polyclonal antibody (anti-IgG-HRP) was tested in the range of 4 to 24 µg/mL. A plateau in signal intensity was observed at 10 µg/mL, which was selected as the optimal concentration, as shown in Figure 2f. Furthermore, it was found that the immunoassay performed better when the incubation of the two antibodies was carried out in a single stage, reducing the overall assay time (Figure 2g) (B/S ratio—where the blank corresponds to the response at 0 µg/mL of FB1 and the signal to the response at 10 µg/mL of FB1).

#### 3.1.5. Evaluation of Immunoreaction

The immunoreaction time between BSA-FB, mAb-FB and anti-IgG-HRP was optimized by testing reaction times ranging from 15 to 90 min. The signal increased up to 60 min, at which point the highest signal was observed, making 60 min the optimal reaction time (Figure 2h).

The temperature and incubation time for the free antigen (10 µg/mL of FB1), mAb-FB and anti-IgG-HRP were optimized. The optimal incubation time was 15 min, and the optimal temperature was 20 °C, as shown in Figure 2i,j.

#### 3.1.6. Evaluation of Enzymatic Reaction

The enzymatic reaction time, in the absence of light, was evaluated at different time intervals (0.5, 1, 1.5, 2, 2.5, 3 and 5 min). The highest signal-to-noise ratio was achieved at 1.5 min, which was selected as the optimal reaction time (Figure 2k).

A summary of all optimized parameters is provided in the Supplementary Material (Table S2).

### 3.2. Fumonisin Determination: Analytical Performance Evaluation of the Immunosensor

**Figure 3:**
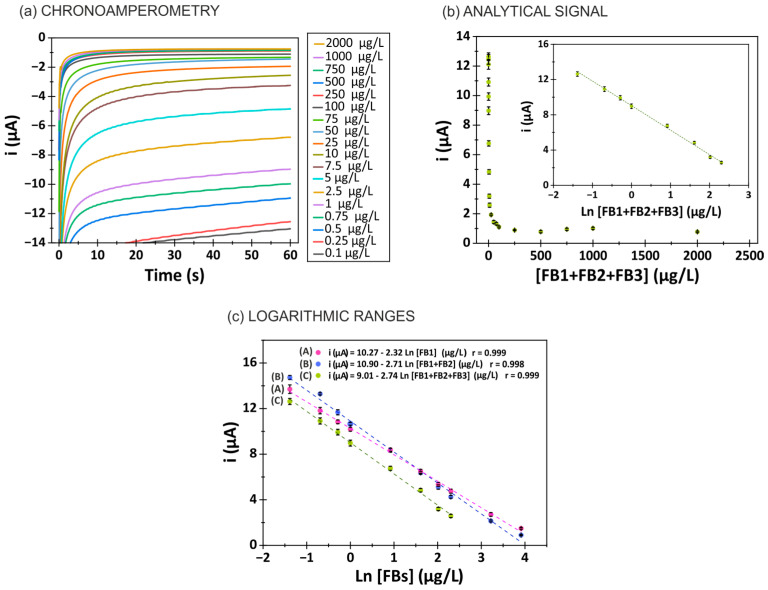
(**a**) Chronoamperograms recorded for increasing concentrations of total fumonisins. Potential: −0.2 V; time: 60 s. (**b**) Relationship between the analytical signal (absolute value of the current recorded at 60 s) and the FB concentration. The inset graph demonstrates the logarithmic range of response. (**c**) Graph of the logarithmic ranges for the different mixtures of fumonisins (FB1, FB1 + FB2 and FB1 + FB2 + FB3) in the measurement buffer (10 mmol/L PBS, pH 7.4; 0.5% BSA).

To evaluate the performance of the developed immunosensor, various concentrations of free antigen (FB1, FB1 + FB2 and FB1 + FB2 + FB3) ranging from 0.1 to 2000 µg/L were tested using the optimized immunosensor. Chronoamperograms obtained for the different concentrations of FB1 + FB2 + FB3 are shown in Figure 3a. As the concentration of free antigen increased, a decrease in the cathodic current of TMB was observed. The electrochemical current generated at 60 s was selected as the analytical signal. Figure 3b illustrates the relationship between total fumonisins (FB1 + FB2 + FB3) and the analytical signal, which follows a logarithmic equation in the range of 0.25 to 10 µg/L. For both FB1 and the FB1 + FB2 mixture, the logarithmic range was 0.25 to 50 µg/L (Figure 3c). The logarithmic relationships and correlation coefficients are as follows:

i (µA) = −2.32 [FB1](µg/L) + 10.27; r = 0.999(1)

i (µA) = −2.71 [FB1 + FB2](µg/L) + 10.90; r = 0.998(2)

i (µA) = −2.74 [FB1 + FB2 + FB3](µg/L) + 9.01; r = 0.999(3)

The limits of detection (LOD = 3 Sb/m) and quantification (LOQ = 10 Sb/m) (Sb: standard deviation of the blank; m: calibration slope [68]) were 0.14 µg/L and 0.47 µg/L for FB1, 0.15 µg/L and 0.50 µg/L for FB1 + FB2 and 0.12 µg/L and 0.39 µg/L for FB1 + FB2 + FB3, respectively.

The system’s reproducibility was assessed by comparing calibration slopes obtained using different sensors prepared under identical conditions on 5 different days, resulting in relative standard deviations (RSDs) of 3% for FB1, 2% for FB1 + FB2 and 2% for FB1 + FB2 + FB3 (see Supplementary Material S3). This inter-day reproducibility demonstrates the method’s robustness over time. For inter-electrode precision, the RSD was calculated using different electrodes tested on the same day under the same conditions (n = 4), yielding an RSD of 1% in all cases.

Table 1 presents a comparison of the analytical characteristics of the developed immunosensor with previously reported electrochemical aptasensors and immunosensors. As shown, the developed immunosensor demonstrates comparable performance to most other systems requiring electrode modification and is the only one reporting total fumonisin determination.

### 3.3. Selectivity and Stability of the Immunosensor

**Figure 4:**
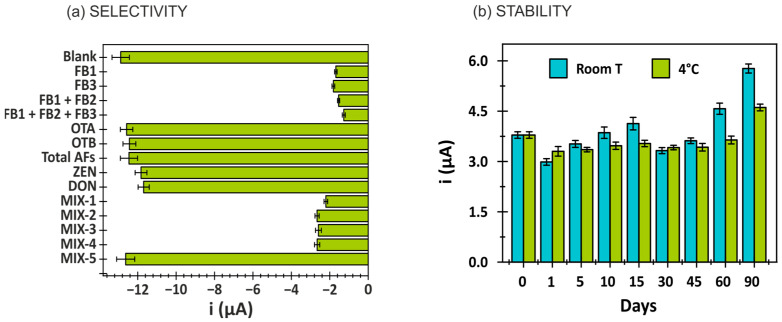
(**a**) Selectivity study: immunosensor response for different mycotoxins (FB1, FB3, FB1 + FB2, FB1 + FB2 + FB3, OTA, OTB, Total AFs, ZEN, DON) individually and in mixtures (MIX-1: FB1, OTA, OTB, Total AFs, ZEN, DON; MIX-2: FB3, OTA, OTB, Total AFs, ZEN, DON; MIX-3: FB1 + FB2, OTA, OTB, Total AFs, ZEN, DON; MIX-4: FB1 + FB2 + FB3, OTA, OTB, Total AFs, ZEN, DON; MIX-5: OTA, OTB, Total AFs, ZEN, DON). (**b**) Stability evaluation: B/S ratio (blank/signal) obtained for immunoassays (FB1 + FB2 + FB3 with concentration of 10 µg/L) performed on different days on BSA-FB-modified electrodes stored both at room temperature and at 4 °C.

The selectivity of the developed immunosensor was assessed by evaluating its response to potential interference from mycotoxins commonly found in food samples. These included ochratoxin A (OTA), ochratoxin B (OTB), total aflatoxins (B1, B2, G1, G2), zearalenone (ZEN) and deoxynivalenol (DON), each tested individually and as a mixture (MIX-5), at concentrations of 200 µg/L. As shown in Figure 4a, no significant variation in the analytical signal was observed for any individual compound or the mixture when compared to the blank. Furthermore, mixtures containing these interfering mycotoxins in combination with a 100 µg/L concentration of fumonisins—FB1, FB3, FB1 + FB2, and FB1 + FB2 + FB3 (denoted as MIX-1, though MIX-4)—did not effect the specific signal corresponding to fumonisins (FB1, FB2, FB3). These results confirm the high selectivity of the developed biosensor for the quantification of total fumonisins (FB1 + FB2 + FB3).

The stability of the immunosensor was evaluated by storing BSA-FB-modified electrodes at both room temperature and 4 °C. Immunoassays were conducted periodically using 0 and 10 µg/L concentrations of total fumonisins. As observed in Figure 4b, the modified electrodes maintained performance for at least 60 days when stored at 4 °C, demonstrating a stable blank-to-signal ratio and indicating adequate long-term storage stability.

### 3.4. Method Validation for Analysis of Fumonisins in Maize Products by LC-MS/MS

Method validation was performed using maize flour, verified to be free of fumonisins (Section 2.2.1), as a blank matrix. Homogenized 5 g portions of this matrix were fortified with a known mixture of fumonisins for analytical validation.

Each batch included a reagent blank and a blank matrix sample to monitor cross-contamination and analytical interference. The validation procedure evaluated key parameters such as linearity, limit of quantification (LOQ), trueness, repeatability (within-laboratory), reproducibility and matrix effects, in accordance with the protocol previously described [69]. Fresh calibration standards were prepared for each analytical run, using a bracketing calibration approach covering a concentration range of 0.2–150 µg/L (equivalent to 1–750 µg/kg). As summarized in the Supplementary Material (Table S4), the method met all validation criteria.

The limit of quantification (LOQ) for the LC-MS/MS method was established at 10 µg/kg, corresponding to the lowest concentration that satisfies the method performance requirements outlined in the Codex Guideline CXG 90-2017 of the validation studies [70].

### 3.5. Evaluation of Matrix Effects in Maize Samples and Naturally Contaminated Tortilla Samples

Due to the absence of a cleanup step in the sample preparation process, residual matrix components remained in the extracts, as evidenced by signals observed in blank samples.

For the LC-MS/MS method, matrix effects (MEs) were estimated by comparing the slopes of calibration curves prepared in maize matrix extracts versus those prepared in pure solvent [71]. The observed MEs were minimal for FB2 (11%) and FB3 (13%), and moderate for FB1 (32%) [72]. Given the relatively low impact of MEs, external calibration using standards prepared in a generic solvent (acetonitrile/methanol/water, 25:25:50 *v*/*v*/*v*) was deemed suitable.

**Figure 5:**
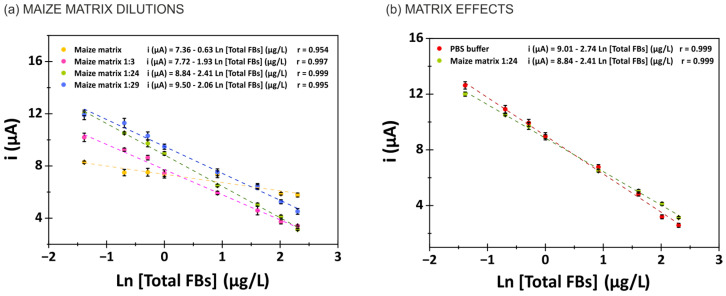
(**a**) Study of different dilutions of the maize matrix in buffer solution (10 mmol/L PBS pH 7.4; 0.5% BSA): without dilution (yellow), maize matrix dilution 1:3 ratio (pink), maize matrix dilution 1:24 ratio (green) and maize matrix dilution 1:29 ratio (purple). (**b**) Matrix effect study. Relationship between the analytical signal and total FBs concentration in buffer solution (10 mmol/L PBS pH 7.4; 0.5% BSA) (green) and maize matrix diluted with buffer (1:24 ratio) (red).

For the immunosensor, matrix effects were evaluated using the standard addition method in the maize matrix diluted to a 1:24 ratio in the buffer. Blank maize samples were spiked, extracted and serially diluted to obtain increasing concentrations of total fumonisins (FB1 + FB2 + FB3) in the range of 0.1–50 µg/L. Signals from matrix extracts were compared to those of standards in PBS buffer to quantify MEs. A matrix effect of 12% was observed, considered negligible. To further minimize the matrix’s influence, different dilution factors were tested in PBS buffer (10 mmol/L PBS pH 7.4; 0.5% BSA). Figure 5a demonstrates that a 1:24 dilution offered optimal immunosensor response and was selected as the standard dilution to minimize the matrix effects. Figure 5b shows that while the presence of the matrix slightly reduces the calibration slope, the effect is sufficiently small to permit reliable analysis. As matrix effects cannot be entirely eliminated, the use of matrix-matched calibration is recommended for accurate quantification of total fumonisins in maize using the immunosensor.

As shown in Table 2, the immunosensor demonstrated excellent recovery values—calculated from the matrix-matched calibration curve: i (µA) = −2.41 [Total FBs] + 8.84—ranging from 95% to 103%. The LOD and LOQ in maize matrix were determined to be 6.07 µg/kg (0.12 µg/L) and 20.25 µg/kg (0.40 µg/L), respectively. These values are significantly below both the European regulatory limit for infants and children (200 µg/kg) and the maximum allowable limit for maize-based foods intended for the general population (1 mg/kg). These findings position the immunosensor as a highly suitable tool for the detection of total fumonisins in maize and tortilla samples.

Finally, naturally contaminated maize tortilla samples were analyzed for total fumonisin content, and the results were compared to those obtained via the validated LC-MS/MS method (see Table 3).

### 3.6. Cross-Validation of Immunosensor Performance with LC-MS/MS

**Figure 6:**
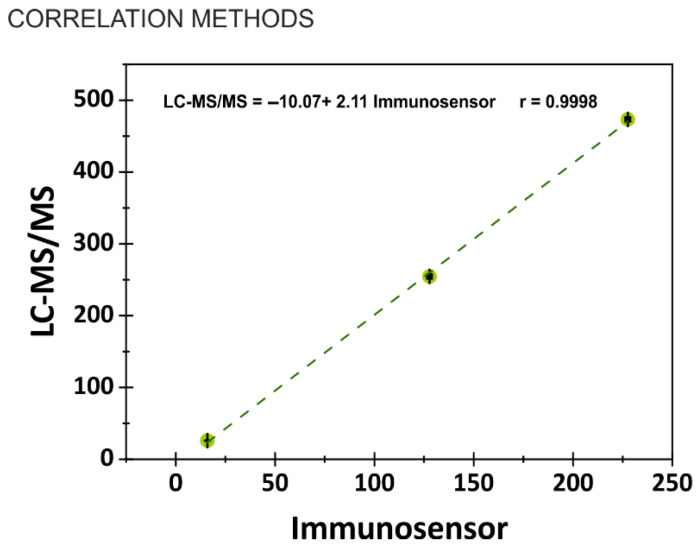
Correlation between LC-MS/MS and the electrochemical immunosensor.

To validate the analytical performance of the developed immunosensor, a comparative analysis was conducted against the reference LC-MS/MS method. Both external calibration and a matrix-matched calibration approaches were employed, and identical sample extracts were analyzed using both techniques. As illustrated in Figure 6, an excellent correlation was observed between the two methods (r = 0.996), indicating the absence of systematic errors in the immunosensor results. This strong correlation confirms the reliability and accuracy of the immunosensor. Notably, the LOQ achieved with the electrochemical immunosensor is comparable to that of the LC-MS/MS method.

## 4. Conclusions

An electrochemical immunosensor targeting the total fumonisin content (FB1 + FB2 + FB3) has been successfully developed utilizing screen-printed carbon electrodes. The biosensor exhibits excellent analytical characteristics, including a broad dynamic range, low detection limit, high reproducibility, remarkable selectivity and long-term stability. Its analytical performance has been rigorously validated through direct comparison with LC-MS/MS, demonstrating outstanding agreement between both methods.

Additionally, a simplified sample preparation protocol was established for the extraction of free fumonisins from maize-derived products, involving a straightforward solvent extraction followed by dilution. By employing either buffer-based or matrix-matched calibration strategies, matrix effects were effectively mitigated, as reflected by the high recovery rates obtained in both fortified samples and naturally contaminated tortilla samples from Belize.

In summary, the developed immunosensor presents a user-friendly, cost-effective and rapid alternative to conventional techniques such as LC-MS/MS and ELISA, while maintaining comparable sensitivity. With an LOQ well below the regulatory limits established for infant and child food safety, the sensor holds significant promise for monitoring fumonisin contamination in sensitive food matrices, including baby foods—where even trace amounts may pose serious health risks. The main advantages of the developed immunosensor are related to its applicability to point-of-need testing due to its simplicity, low cost and capability to detect total fumonisins in real samples with minimal sample preparation. While the analytical performance is comparable to existing methods, the novelty lies in the total fumonisin detection and its direct application to minimally processed maize-based products without the need for extraction kits or electrode modification.

Importantly, the detailed methodological descriptions and validation protocols presented in this study provide a practical framework for laboratories aiming to implement immunosensor-based screening approaches. To facilitate this technology’s adoption, a video demonstrating the immunosensor’s procedure is available as supplementary material.

## Figures and Tables

**Table 2:** Immunosensor levels of recovery. Extracts were fortified at 30, 300 and 600 µg/kg as the sum of FB1 + FB2 + FB3, and the analytical signals were compared with those obtained with the matrix-matched calibration (average of measurements of 3 different days, n = 4).

Sample	Fortification Level of Total FBs (µg/kg)	Nominal Concentration ^a^ (µg/L)	Current Matrix ^b^ (µA)	Current in the Sample Extract (µA)	Measured Concentration (µg/L) ± CV %	Recovery
Maize	30	15	−10.20 ± 0.10	−10.26 ± 0.08	14.26 ± 4%	95%
	300	150	−4.47 ± 0.10	−4.54 ± 0.07	154.33 ± 4%	103%
	600	300	−2.84 ± 0.07	−2.95 ± 0.04	302.95 ± 3%	101%

^a^ Nominal concentration of FBs in extract after sample pre-treatment (5 g of fortified sample, 2 mL of water and 10 mL of extraction solution). ^b^ Matrix standard addition signal.

**Table 3:** Determination of total fumonisins in naturally contaminated tortilla samples using the validated LC-MS/MS method and the developed electrochemical immunosensor (n = 4).

Tortilla Sample	LC-MS/MS (Validated Method) (µg/kg)	Electrochemical Immunosensor (µg/kg)	Relative Error ^a^
S-4	84.0	79.5	−5.4%
S-295	180.4	174.2	−3.4%
S-296	210.3	196.3	−6.7%
S-306	119.9	123.1	+2.7%
S-311	114.6	118.0	+3.0%

^a^ Relative error: calculated using the concentrations measured with LC-MS/MS and those of the proposed method.

## Data Availability

Data discussed in this work are available in the Supplementary Material section.

## Supplementary Materials

The following supporting information can be downloaded at https://www.mdpi.com/article/10.3390/bios15080526/s1: Table S1: Optimization of MS parameters; Table S2: Optimization of biosensor parameters; Table S3: Reproducibility of the fumonisin immunosensors; Table S4: LC-MS/MS method validation results; Video S1: Sensor preparation and results; Video S2: Maize sample preparation. References [73,74] are cited in the supplementary materials.

## Abbreviations

The following abbreviations are used in this manuscript:

SPCE	Screen-Printed Carbon Electrode
FB	Fumonisin
IARC	International Agency for Research on Cancer
HPLC-MS	High-Performance Liquid Chromatography–Mass Spectrometry
GC-MS	Gas Chromatography–Mass Spectrometry
TLC	Thin-Layer Chromatography
ELISA	Enzyme-Linked ImmunoSorbent Assays
LC-MS/MS	Liquid Chromatography–Tandem Mass Spectrometry
ZEN	Zearalenone
OTA	Ochratoxin A
OTB	Ochratoxin B
DON	Deoxynivalenol
AF	Aflatoxins
BSA	Bovine Serum Albumin
TMB	3,3′-5,5′-Tetramethylbezidine
PBS	Phosphate-Buffered Saline solution
BSA-FB	Fumonisin antigen conjugate
mAb-FB	Mouse monoclonal antibody specific to Fumonisin
Anti-IgG-HRP	Polyclonal rabbit anti-mouse IgG-HRP
HRP	Horse Radish Peroxidase
ESI	Electrospray ionization
MRM	Multiple Reaction Monitoring
LOD	Limit of Detection
LOQ	Limit of Quantification
SB	Standard Deviation
RSD	Relative Standard Deviation
ME	Matrix Effect
