# Supplementary material for: Fumonisin B Determination in Maize Products from Belize Using an Immunosensor Based on Screen-Printed Carbon Electrodes
Source: Biosensors (Basel). 2025 Aug 12;15(8):526. doi: 10.3390/bios15080526 (PMC12384066; doi:10.3390/bios15080526)
Supplement: Supplementary file 1 [file biosensors-15-00526-s001.zip › De la Escosura-Muniz_Fumonisins_Supplementary Material.pdf]

Supplementary Information

Fumonisins B determination in maize products using an immunosensor based on screen-printed carbon electrodes

Beatriz Pérez-Fernández[1,2], Britt Marianna Maestroni[2], Carlotta Cozzani[2], Colette Eusey[3], Natalie Gibson[3], Alfredo de la Escosura-Muñiz[1,4*] and Christina Vlachou[2]

1 NanoBioAnalysis Group - Department of Physical and Analytical Chemistry, University of Oviedo, Julián Clavería 8, 33006, Oviedo, Spain
2 Food Safety and Control Laboratory, Joint FAO/IAEA Centre of Nuclear Techniques in Food and Agriculture, Department of Nuclear Sciences and Applications, International Atomic Energy Agency, Wagramerstrasse 5, A-1400, Vienna, Austria
3 Belize Agriculture Health Authority, Central Investigation Laboratory, Belize City, Belize
4 Biotechnology Institute of Asturias, University of Oviedo, Santiago Gascon Building, 33006 Oviedo, Spain
* Correspondence: alfredo.escosura@uniovi.es

Index of Supplementary Material

S1. Optimization of MS parameters.

S2. Optimization of biosensor parameters.

S3. Reproducibility of the immunosensor.

S4. LC-MS/MS method validation results.

S5. Video on 'Sensors preparation and results' (see attached file).

S6. Video on 'Maize samples preparation' (see attached file).

S1. Optimization of MS parameters.

The MS parameters and the collision energies were optimized to achieve the highest responses for all FBs. The optimized transitions, the collision energies and retention times of each FB are presented in **Table S1**.

Table S1. The MRM transitions and the collision energies (CE) for the fumonisins FB1, FB2 and FB3 using the UHPLC Column: Waters Acquity BEH C8, 2.1mm x 100 mm, 1.7 μm, coupled with Acquity UPLC BEH C8 VanGuard pre column (Waters, USA)

Analyte	RT (min)	Transition 1	CE (V)	Transition 2	CE (V)	Transition 3	CE (V)
FB1	7.6	722.4>334.1	-43	722.4>352.1	-37	722.4>704.4.	-29
FB2	8.3	706.4> 336.1	-39	706.40> 318.1	-40	706.4>354.1	-35
FB3	8.0	706.4> 336.1	-39	706.40> 318.1	-40	706.4>354.1	-35
^{13}C34 FB1	7.5	756.4>374.4	-30	756.4>392.3	-30	756.4>356.3	-40

S2. Optimization of biosensor parameters.

Table S2. Summary of all optimized parameters for the development of an immunosensor for the determination of total fumonisins in maize products.

Parameter	Optimal value
[BSA-FB]	25 µg/mL
Incubation time BSA-FB	90 min
Blocking step	0.5% w/v BSA, PBS
Blocking time	20 min
[mAb-FB]	2 µg/mL
[anti IgG-HRP]	10 µg/mL
Nº. competitive incubation steps	1 step
Competitive incubation time	60 min
Pre-incubation time	15 min
Pre-incubation temperature	20°C
Enzymatic reaction time	1.5 min
Measurement technique	Chronoamperometry
Potential	-0.2 V
Measurement time	60 s

S3. Reproducibility of the fumonisins immunosensors.

Table S3. Reproducibility of the fumonisins immunosensors for the slope of calibrations performed on 5 different days.

Linear regression (FB1)	Correlation coefficient (r)
i (μA) = -2.40 [FB1] (μg/L) + 10.63	0.999
i (μA) = -2.32 [FB1] (μg/L) + 10.27	0.999
i (μA) = -2.24 [FB1] (μg/L) + 10.23	0.997
i (μA) = -2.24 [FB1] (μg/L) + 10.03	0.999
i (μA) = -2.31 [FB1] (μg/L) + 9.70	0.998

Linear regression (FB1+FB2)	Correlation coefficient (r)
i (μA) = -2.70 [FB1+FB2] (μg/L) + 11.62	0.991
i (μA) = -2.85 [FB1+FB2] (μg/L) + 12.59	0.995
i (μA) = -2.74 [FB1+FB2] (μg/L) + 11.69	0.994
i (μA) = -2.72 [FB1+FB2] (μg/L) + 10.91	0.995
i (μA) = -2.71 [FB1+FB2] (μg/L) + 10.90	0.998

Linear regression (FB1+FB2+FB3)	Correlation coefficient (r)
i (μA) = -2.65 [FB1+FB2+FB3] (μg/L) + 8.70	0.998
i (μA) = -2.74 [FB1+FB2+FB3] (μg/L) + 9.15	0.998
i (μA) = -2.75 [FB1+FB2+FB3] (μg/L) + 9.29	0.998
i (μA) = -2.74 [FB1+FB2+FB3] (μg/L) + 9.01	0.999
i (μA) = -2.74 [FB1+FB2+FB3] (μg/L) + 8.45	0.997

S4. LC-MS/MS method validation results.

This appendix describes the validation of a method for the determination of fumonisins in maize samples by LC-MS/MS. This method was used for the determination of the fumonisins FB1, FB2 and FB3 at 10, 100 and 200 μg/kg in maize matrix. The fortification levels were selected to cover concentrations at or below the maximum permitted levels (MLs) of the target analytes, being the maximum levels for the sum of FB1 and FB2 in the EU legislation for processed maize based foods and baby foods for infants and young children.

Blank maize samples were fortified with a mixture of fumonisins standards at 3 concentration levels (10, 100 and 200 μg/kg), 5 replicate analytical portions were generated at each level, and the study was repeated on 3 different days to estimate the within laboratory reproducibility of the method. The performance criteria established in Commission Implementing Regulation (EU) 2023/2782 [1] were used as a basis for method validation and the guidance document on analytical quality control and validation procedures for pesticide residues analysis in food and feed SANTE/2019 was used as a guidance for the analytical calibration [2]. The linearity of the FLC-MS/MS measurement was established through a set of calibration standards in matrix covering a concentration range from 0.2 to 150 μg/L (1 – 750 μg/kg). The identification of analytes was based on retention time and ion ratio of coinciding peaks for at least two diagnostic transitions in the correct abundance ratio. At least two diagnostic multiple reaction monitoring transitions (MRMs) were selected for each analyte; the ion ratios of the two diagnostic MRMs of the analytes in the spiked samples were compared to the reference ion ratios of the calibration standards. The differences were considered acceptable when scoring within ± 30%.

The recovery results, the calculated repeatability and reproducibility within laboratory standard deviation, are shown in **Table S4**. The LOQ of the method for each aflatoxin is set at the lowest fortification level. As it is demonstrated in **Table S4**, the performance criteria for analytical methods for mycotoxins laid down in the Commission Implementing Regulation (EU) 2023/2782 are met for all analytes.

Table S4. The scope of the method, the established LOQ and the within laboratory repeatability and reproducibility values, expressed as relative standard deviation (%), at levels of each FBs of 10, 100 and 200 μg/kg in maize matrix (n = 15 for each FB and each fortification level).

		Fortification level 10 μg/kg			Fortification level 100 μg/kg			Fortification level 200 μg/kg		
**Analytes**	**LOQ (μg/kg)**	**Average Recovery R%**	**Repeatability RSD r%**	**Reproducibility RSD R%**	**Average Recovery R%**	**Repeatability RSD r%**	**Reproducibility RSD R%**	**Average Recovery R%**	**Repeatability RSD r%**	**Reproducibility RSD R%**
FB1	10	106.2	0.6	9.6	106.1	1.7	4.3	100.1	4.3	4.4
FB2	10	75.3	0.2	5.1	69.4	1.8	9.0	64.3	3.3	9.4
FB3	10	78.2	0.4	9.6	79.0	1.9	7.0	72.2	4.7	5.8

As a conclusion, the method was successfully validated for the determination of fumonisins in maize samples by LC-MS/MS.

References:

1. European Commission Commission implementing regulation (EU) 2023/2782 of 14 December 2023 laying down the methods of sampling and analysis for the control of the levels of mycotoxins in food and repealing Regulation (EC) No 401/2006. *Off. J. Eur. Union* **2023**, *2782*, 1–44.
2. SANTE/12682/2019 Guidance document on analytical quality control and method validation for pesticide residues analysis in food and feed. *Saf. Food Chain Pestic. Biocides. Eur. Comm.* **2019**, 1–48.
